# Relationship between Obstructive Sleep Apnea Severity and Sleep, Depression and Anxiety Symptoms in Newly-Diagnosed Patients

**DOI:** 10.1371/journal.pone.0010211

**Published:** 2010-04-16

**Authors:** Paul M. Macey, Mary A. Woo, Rajesh Kumar, Rebecca L. Cross, Ronald M. Harper

**Affiliations:** 1 School of Nursing, David Geffen School of Medicine at UCLA, University of California Los Angeles, Los Angeles, California, United States of America; 2 Brain Research Institute, David Geffen School of Medicine at UCLA, University of California Los Angeles, Los Angeles, California, United States of America; 3 Department of Neurobiology, David Geffen School of Medicine at UCLA, University of California Los Angeles, Los Angeles, California, United States of America; RAND Corporation, United States of America

## Abstract

Obstructive sleep apnea (OSA) occurs in at least 10% of the population, and leads to higher morbidity and mortality; however, relationships between OSA severity and sleep or psychological symptoms are unclear. Existing studies include samples with wide-ranging comorbidities, so we assessed relationships between severity of OSA and common sleep and psychological disturbances in recently diagnosed OSA patients with minimal co-morbidities. We studied 49 newly diagnosed, untreated OSA patients without major co-morbidities such as mental illness, cardiovascular disease, or stroke; subjects were not using psychoactive medications or tobacco (mean ± std age: 46.8±9.1 years; apnea/hyponea index [AHI]: 32.1±20.5 events/hour; female/male: 12/37; weight <125 kg). We evaluated relationships between the AHI and daytime sleepiness (Epworth Sleepiness Scale; ESS), sleep quality (Pittsburg Sleep Quality Index; PSQI), depressive symptoms (Beck Depression Inventory-II; BDI), and anxiety symptoms (Beck Anxiety Inventory; BAI), as well as sex and body mass index (BMI). AHI was similar in females and males. Mean levels of all symptoms were above normal thresholds, but AHI was not correlated with age, ESS, PSQI, BDI, or BAI; only BMI was correlated with OSA severity. No differences in mean AHI appeared when subjects were grouped by normal versus elevated values of ESS, PSQI, BDI, or BAI. Consistent with other studies, a strong link between OSA severity and psychological symptoms did not appear in these newly diagnosed patients, suggesting that mechanisms additional to the number and frequency of hypoxic events and arousals occurring with apneas contribute to adverse health effects in OSA. OSA patients presenting with mild or moderate severity, and no major co-morbidities will not necessarily have low levels of sleep or psychological disturbances.

## Introduction

Obstructive sleep apnea (OSA) occurs in approximately 10% of the population, leads to physical and psychological co-morbidities, and is associated with higher all-cause mortality [1], [2], [3], [4]. Symptoms of OSA include daytime sleepiness, poor sleep quality, and co-morbidities such as high levels of depressive symptoms and anxiety [5], [6], [7], but the relationship between OSA severity and accompanying symptoms is weak. The severity of OSA, as defined by the American Academy of Sleep Medicine [4], is classified by the number of partial or complete cessations of breathing per hour during sleep, the apnea/hypopnea index (AHI). However, most studies do not report strong relationships between AHI and severity of symptoms [8], [9]. The extent to which a patient's reported AHI level predicts severity of symptoms remains unclear, and is the topic of recent investigations [10], [11], [12], [13].

Excessive daytime sleepiness is the most common symptom associated with OSA [4], [6]. High daytime sleepiness is often a primary reason for referring a person for a polysomnographic study, and the evidence of higher measures of daytime sleepiness in OSA is unequivocal [4], [6]. Since OSA is defined according to a cut-off in AHI, a logical assumption is that higher AHI leads to higher daytime sleepiness. However, while some large samples show a statistically significant correlation, AHI and daytime sleepiness measures are not strongly correlated [9], [13], [14], [15], [16], [17], a pattern noted in the revised International Classifications of Sleep Disorders criteria [18].

Other symptoms that are present in persons with OSA also show only weak relationships with AHI. Poor sleep quality is common in people with OSA, but is not explained by AHI [19], even though obstructive events are typically associated with arousals. Apneic indices fail to explain other characteristics: levels of depressive symptoms, while high in approximately half of OSA patients [5], [20], [21], [22], correlate at best poorly with AHI [10], [23], and similar findings are true for anxiety symptoms [24], [25], [26]. Mood and affect disturbances could arise from neural injury that occurs in the condition [27], [28], [29], [30], and such injury would be expected to be greater with increasing severity, since animal evidence shows that repeated hypoxic episodes lead to cellular injury and death in a dose-dependent fashion [31], [32], [33], [34].

Existing studies mostly describe typical OSA populations that include patients with major risk factors for illness and co-morbidities, such as severe obesity, tobacco use, mental disorders, and major cardiovascular disease. Since these factors influence sleep and affective symptoms, it is possible that a stronger relationship between OSA severity and symptoms will be present in a sample of patients without major health issues other than OSA. Therefore, our objective was to describe relationships between severity of OSA and severity of common sleep and psychological disturbances present in the sleep disorder, in a sample with minimal co-morbidities. We studied recently-diagnosed, moderate to severe OSA patients with minimal comorbidities, who had not started treatment, and who did not have other major cardiovascular or psychiatric disorders.

## Methods

All procedures were approved by the UCLA Institutional Review Board, and all participants provided written, informed consent.

We performed a cross-sectional study of OSA patients, diagnosed based on an overnight sleep study, who completed commonly-used questionnaires for evaluation of sleep and psychological symptoms.

### Subjects

We studied 49 newly diagnosed, untreated OSA patients without major comorbidities (Table 1). Subjects were recruited via the UCLA Sleep Disorders Laboratory. Patients were untreated for the sleep disorder, did not currently smoke, and were not taking psychiatric medications. Screening was by self-report on initial clinical interview prior to study onset. These subjects were originally recruited as part of a brain imaging study [28], so inclusion criteria due to scanner limitations included a weight of less than 125 kg, no claustrophobia, and absence of metallic implants.

**Table 1:** Subject information.

*Characteristics*				
	Sex 12 ♀: 37 ♂			
		Mean	St Dev	Range
	Age (years)	**46.8**	**±9.1**	**31–63**
	BMI (kg/m^2^)	**30.4**	**±5.1**	**21–43**
	Years Education* (N = 11)	**19**	**±3**	**16–25**
*Sleep*				
	AHI (events/hour)	**32.1**	**±20.5**	**5–101**
	SaO_2_ nadir†	**80.2**	**±9.4**	**50–96**
	SaO_2_ baseline†	**94.9**	**±1.9**	**88–97**
*Apnea type* †			**N**	**%**
	Positional (supine)		**8**	**17%**
	REM-only		**11**	**23%**
	All-stage (non-specific)		**29**	**60%**
*Symptoms*				
	ESS	**10.5**	**±4.9**	**0–24**
	PSQI	**9.3**	**±4.2**	**0–20**
	BDI	**9.7**	**±8.8**	**0–37**
	BAI	**11.2**	**±12.1**	**0–54**
*Medical History*			**N**	**%**
	Cardioascular disease (angina, atherosclerosis, cerebrovascular disease, coronary artery disease, heart failure, myocardial infarction)		**0**	**0%**
	Diabetes		**5**	**10%**
	Gout		**2**	**4%**
	Hypertension		**17**	**35%**
	Diagnosed Mental Health Disorder		**0**	**0%**
	Migraines		**0**	**0%**
	Sleep disorder (not OSA)		**0**	**0%**
	Stroke		**0**	**0%**
	Tobacco (past use)		**8**	**16%**
	Vascular congenital abnormality		**0**	**0%**
*Medications*				
	Anti-depressant (past use)		**3**	**6%**
	Anti-anxiety (past use)		**1**	**2%**
	Statin†		**9**	**19%**
	ACE/ARB†		**6**	**13%**

Characteristics and symptoms of 49 OSA patients; BMI  =  body mass index; AHI  =  apnea/hypopnea index (severity of OSA); SaO_2_  =  blood oxygen saturation (oximetry); REM  =  rapid eye movement sleep; ESS  =  Epworth Sleepiness Scale; PSQI  =  Pittsburgh Sleep Quality Index; BDI  =  Beck Depression Inventory II; BAI  =  Beck Anxiety Inventory; ACE  =  angiotensin-converting enzyme inhibitor; ARB  =  angiotensin receptor blocker; statin  =  cholesterol-lowering via inhibition of HMG-CoA reductase enzyme.

*Missing data for N  =  38 subjects; years of education were not originally collected.

† Missing data points (indicates that one subject's value is missing, but not always the same subject); one subject missing for each of SaO_2_ and position measurements due to technical failures, blood pressure medication history not collected for one subject; summary values are shown for the remaining 48 subjects.

### Data Collection

The OSA severity was determined based on an overnight sleep study. The AHI, or number of apnea/hypopnea per hour, was determined according to standard criteria [4].

We collected subject characteristics of age, sex and body mass index (BMI), as well as measures of sleep, depression and anxiety symptoms. All symptoms were assessed with commonly-used self-report questionnaires. Daytime sleepiness was measured with the Epworth Sleepiness Scale (ESS), sleep quality by the Pittsburgh Sleep Quality Index (PSQI), depressive symptoms with the Beck Depression Inventory II (BDI), and anxiety symptoms with the Beck Anxiety Inventory (BAI). These measures show moderate to strong validity and reliability in healthy people and in groups with medical conditions [35], [36], [37], [38], [39], although OSA populations have not been assessed.

### Statistical analysis

The non-parametric Spearman's rho was calculated for AHI correlations with ESS, PSQI, BDI, BAI and BMI. Mean AHI values were compared between groups that were defined by classifying subjects into normal and elevated symptom levels, using independent samples t-tests. The standard *p* threshold of 0.05 was used. The 95% confidence intervals for Spearman's rho were calculated using a bootstrap method (500 samples). Correlations and independent samples t-tests were performed with the statistical software SPSS (Chicago, IL), and the confidence intervals were calculated with the bootstrap function in MATLAB 7.8 with the Statistics Toolbox (The Mathworks Inc., Natwick, MA). Power analyses were performed with G*Power 3 [40].

## Results

### Main Findings

The AHI, the “gold standard” of obstructive sleep apnea severity, was not significantly correlated with symptoms of daytime sleepiness, sleep quality, depression, or anxiety. Specifically, AHI showed no significant correlations with age, ESS, PSQI, BDI, or BAI (Table 2, 1^st^ column). Only BMI showed a significant positive correlation with AHI. Additionally, we found no group differences in mean AHI when subjects were categorized into two groups according to normal/elevated measures of each of ESS, PSQI, BDI, or BAI (Table 3). We used a cutoff of >9 to classify BDI measures as high, a threshold lower than the typical 14, but a more sensitive level [41], which we previously showed can predict additional neural injury in high relative to low BDI OSA patients [42]. The full correlation table showed additional significant relationships between all combinations of BDI, BAI and PSQI (Table 2).

**Table 2:** Correlations.

Correlation table							
		AHI	Age	BMI	ESS	PSQI	BDI
Characteristics							
Age	r	−0.06					
	***p***	***0.67***					
	**CI**	**[−0.33, 0.28]**					
BMI	r	0.33 *	0.07				
	***p***	***0.002***	***0.63***				
	**CI**	**[0.00, 0.57]**	**[−0.29, 0.37]**				
Symptoms							
ESS	r	−0.18	−0.16	0.04			
	***p***	***0.21***	***0.28***	***0.80***			
	**CI**	**[−0.47, 0.36]**	**[−0.47, 0.11]**	**[−0.26, 0.40]**			
PSQI	r	0.07	−0.20	0.12	0.08		
	***p***	***0.61***	***0.16***	***0.40***	***0.56***		
	**CI**	**[−0.21, 0.36]**	**[−0.48, 0.07]**	**[−0.16, 0.41]**	**[−0.23, 0.34]**		
BDI	r	0.03	−0.18	0.13	0.20	0.74 *	
	***p***	***0.83***	***0.23***	***0.36***	***0.16***	***<0.001***	
	**CI**	**[−0.25, 0.34]**	**[−0.49, 0.15]**	**[−0.16, 0.42]**	**[−0.06, 0.50]**	**[−0.57, 0.85]**	
BAI	r	0.09	0.02	0.21	0.24	0.47 *	0.72 *
	***p***	***0.55***	***0.91***	***0.14***	***0.09***	***<0.001***	***<0.001***
	**CI**	**[−0.17, 0.38]**	**[−0.28, 0.33]**	**[−0.08, 0.48]**	**[−0.04, 0.44]**	**[−0.22, 0.67]**	**[−0.54, 0.82]**

Correlation table of subject characteristics, symptoms, and OSA severity; Spearman's rho, p value, and 95% confidence interval (CI) are shown for each correlation (* indicates significant). BMI  =  body mass index; AHI  =  apnea/hypopnea index (severity of OSA); ESS  =  Epworth Sleepiness Scale; PSQI  =  Pittsburgh Sleep Quality Index; BDI  =  Beck Depression Inventory II; BAI  =  Beck Anxiety Inventory.

**Table 3:** Mean comparisons.

AHI – Comparing means									
			N	Mean ± std	N	Mean ± std	Diff	se	*P*
Characteristics									
				Female		Male	♂ - ♀		
	Sex		**12**	**24.3±18.8**	**37**	**34.1±19.7**	**9.9**	**6.3**	**0.8**
Symptoms		Cutoff		Normal		High	High -Normal		
	PSQI	>5	**7**	**22.7±13.0**	**42**	**33.2±20.3**	**10.5**	**5.8**	**0.2**
	ESS	>9	**19**	**32.2±14.8**	**30**	**31.4±22.5**	**−0.8**	**5.8**	**0.9**
	BDI	>9	**29**	**33.0±20.9**	**20**	**29.9±18.2**	**−3.1**	**5.6**	**0.6**
	BAI	>7	**26**	**28.9±16.5**	**23**	**34.9±22.7**	**6.1**	**5.7**	**0.2**

Mean AHI compared across groups of subjects classified according to symptom severity or sex, using independent samples t-tests; ESS  =  Epworth Sleepiness Scale; PSQI  =  Pittsburgh Sleep Quality Index; BDI  =  Beck Depression Inventory II; BAI  =  Beck Anxiety Inventory.

### Power Analysis

A medium-to-large *effect* size, measured as Pearson's *r*, of 0.38 (0.34 one-tailed) would be required to detect any relationship with 80% power at α = 0.05 [40]. The largest symptom and AHI correlation effect size (non-parametric equivalent) found in this sample was 0.18 (Table 2, 1^st^ column).

## Discussion

A strong link between the standard measure of OSA severity, the AHI, and accompanying symptoms did not appear in these newly-diagnosed patients, who were untreated and without major co-morbid illness. However, the presence of OSA, diagnosed primarily based on AHI values, was associated with abnormally high levels of depressive and anxious symptoms, daytime sleepiness, and poor sleep quality. These findings suggest that mechanisms other than the number and frequency of hypoxic events contribute to adverse health effects in this patient population.

As found in other OSA populations [11], [43], levels of daytime sleepiness, poor sleep quality, depression and anxiety were high, with mean levels of these symptoms above normal cutoff thresholds (Table 3). Thus, the absence of correlation between symptoms and disease severity does not result from low levels of symptoms in this set of OSA patients. One possibility is that the population consists of several distinct subgroups, which are affected differently by the apneic periods. Variations in affective and mood symptoms in particular are likely to be associated with variations in a number of symptoms related to neural functioning, as we previously showed that, in subsets of the present 49 OSA patients, depressive symptoms are associated with brain structural changes, as are symptoms of anxiety [42], [44]. Our neuroimaging findings show differential effects on brain structure of diabetes, sleep state in which apneas occur, and sex [45], [46], [47]. The heterogeneous nature of characteristics associated with brain changes in OSA suggests that these variables should be considered separately with regards to their influence on sleep and psychological symptoms; the present study did not have enough subjects to power such tests.

The findings support the hypothesis that AHI is not the most appropriate polysomnographic measure of clinical impact of OSA, a relationship noted previously [48], [49]. One possible reason is that AHI is based on numbers of all apneas and hypopneas, but not severity of apneic events. Symptoms like poor sleep quality and daytime sleepiness may be more closely related to number and extent of arousals, which are principally associated with obstructive apneas. The frequency of arousals is a better predictor of fatigue [8], another common symptom of OSA [50]. Similarly, blood oxygen desaturation appears to be a better predictor than AHI of daytime sleepiness [51] and cardiovascular sequelae [52], [53]. The problem of defining clinically-relevant operational definitions of sleep variables was part of the motivation for the development by the American Academy of Sleep Medicine of new scoring criteria in 2007 [54], although it is unclear whether the new definitions of severity are better predictors of symptoms.

The lack of strong correlations between AHI and subjective sleep symptoms suggests that factors other than respiratory events and arousal contribute to these disturbances. Affective disruptions appear to be closely related to sleep quality: the PSQI had the strongest correlations with the BDI and BAI, similar to a previous report [25]. While self-reports are potentially inaccurate, especially in psychologically disturbed populations, the PSQI has moderate to high validity and reliability [35], with validation in other sleep-disturbed populations [55].

Determining the extent to which mood disruptions are independent of OSA is problematic, since several mental illnesses, including depression, are classified by symptoms that include sleep disturbances [56]. The BDI includes questions on symptoms that overlap typical OSA characteristics, such as sleepiness and fatigue, loss of libido, and impaired concentration. This overlap does not alter the depressive symptoms themselves but confounds interpretation of the source of the mood disturbances; we cannot disentangle the extent to which individuals with depressed mood experience a disorder independent of OSA. The DSM-IV criteria for organic rule-out of a primary mood disorder, i.e., diagnosis of a mood disorder that is secondary to a physical medical illness [57], do not clarify whether depressed mood in OSA should be considered secondary to the physical disorder. Our earlier work demonstrating that mildly depressed or anxious OSA subjects show greater brain injury over patients with normal mood or affect suggests a neural basis for the mood disturbances [42], [44]. However, whether OSA preceded the neural injury and mood symptoms cannot be determined in the present cross-sectional study.

Limitations of the current study include the specific nature of the study population; a group with minimal co-morbidities, recent diagnosis, and limited BMI is not representative of the general OSA population. Furthermore, the heterogenous nature of the disorder, even within the restricted sample used here, means that a much larger number of subjects than 49 would need to be studied to verify that the findings generalize to a larger population. Additional limitations relate to the lack of objective measures of symptoms. However, the variables assessed in the present study reflect information typically available to, or easily collected by clinicians. More-accurate measurements may allow relationships between OSA severity and symptoms to be detected, but given the minimal effects found in this and other studies, it is unlikely that any strong correlation would appear using different measures. Other potential approaches to refining the analysis include increasing the number of subjects, or selecting a different OSA population. In particular, a possibility would be to study separate populations based on depression, sex, or sleep quality, to potentially elucidate relationships within those subgroups.

### Clinical Implications

A patient's reported OSA severity should not be assumed to directly relate to severity of accompanying psychological symptoms. These findings do not negate the importance of sleep-disordered breathing; many clinical symptoms are associated with sleep disturbances, and screening for the presence of sleep disorders, including OSA, is important [58]. The present results are limited to newly-diagnosed, untreated, middle-aged OSA patients without major co-morbid illness, and weighing less than 125 kg, so different patterns may appear in other OSA populations. Obesity (measured as BMI), a known risk factor for the sleep disorder, is associated with OSA severity, as is the most prominent co-morbidity, hypertension [12], [59]. For patients who have received a sleep study, the presence of OSA of any severity would suggest that a patient likely has abnormally high levels of daytime sleepiness, depression, and anxiety, as well as poor sleep quality. For patients without an assessment for OSA, but presenting with sleepiness, depression, and complaints of poor sleep, the presence of OSA may be suspected, but the level of symptoms should not be assumed to relate to the severity of the sleep disorder.
